# Visualizing Current-Dependent Morphology and Distribution of Discharge Products in Sodium-Oxygen Battery Cathodes

**DOI:** 10.1038/srep24288

**Published:** 2016-04-12

**Authors:** Daniel Schröder, Conrad L. Bender, Markus Osenberg, André Hilger, Ingo Manke, Jürgen Janek

**Affiliations:** 1 Physikalisch-Chemisches Institut, Justus-Liebig-Universität Giessen, Heinrich-Buff-Ring 17, 35392 Giessen, Germany; 2 Helmholtz-Zentrum Berlin für Materialien und Energie GmbH, Hahn-Meitner-Platz 1, 14109 Berlin, Germany

## Abstract

Synchrotron X-ray tomography and scanning electron microscopy were applied to elucidate the spatial distribution of discharge product (NaO_2_) in the carbon cathode of sodium-oxygen batteries. Various batteries were discharged galvanostatically and their cathodes were analyzed. We observe a particle density gradient along the cathode that scales with the current density applied. Besides, we show that the particle size and shape of discharge product strongly depend on current density, and on whether the particles are deposited close to the oxygen reservoir or near the separator. We correlate our findings to transport limitations for the supplied oxygen and gain crucial information for optimal operation of sodium-oxygen batteries. Our findings imply that for low current densities pore clogging might occur, and that for elevated current densities small high surface area particles with limited electric conductivity form; both phenomena can decrease the available discharge and charge capacity significantly.

Sodium-oxygen batteries (Na/O_2_ batteries) are attractive candidates for future energy storage applications from the theoretical point of view, but fail so far to fulfill the optimistic expectations^1^. Up to now, their practical energy density and cycling stability are deficient^2^,^3^. Despite continuous progress^4^,^5^, fundamental understanding of the electrode reactions^6^ and the formation of reaction products is still limited^7^. Thus, deeper insight is needed to propel development of Na/O_2_ batteries.

The cell reactions during discharge of Na/O_2_ batteries are as follows^2^: Oxygen is reduced at an active site of the positive electrode (cathode) to form superoxide 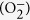, peroxide 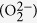, or oxide (O^2−^) species that, together with the cation (Na^+^) from the pure Na metal applied as anode, may form a solid metal oxide phase in the cathode structure^4^,^8^. So far both NaO_2_^3^,^4^,^5^,^7^,^8^,^9^,^10^,^11^ and Na_2_O_2_^12^,^13^,^14^,^15^ have been experimentally reported as discharge product in Na/O_2_ batteries. It appears that the nature of the electrolyte and the water concentration in the cell might determine whether NaO_2_ or Na_2_O_2_ is formed^6^,^11^,^16^.

While the nature of the discharge product in Na/O_2_ batteries is discussed intensively^6^,^11^,^16^,^17^, little is known about the spatial distribution of discharge product in the pores of the cathode layers. A parallel for the product distribution might be drawn from latest research on Li/O_2_ batteries: Model-based^18^ and X-ray photon spectroscopy^19^,^20^ investigations have shown that the primary discharge product, lithium peroxide (Li_2_O_2_), is deposited near the battery separator first, while unwanted lithium carbonate species (Li_2_CO_3_), formed from impurities or in electrolyte decomposition reactions, are deposited near the oxygen reservoir (i.e. at the top of the cathode). In addition, toroidal Li_2_O_2_ particles were visualized by various methods, such as scanning X-ray transmission microscopy^21^ and high-resolution transmission electron microscopy^22^.

On the one hand, Li_2_O_2_ can limit battery performance at elevated current densities due to its poor electric conductivity, and on the other hand Li_2_CO_3_ can cause pore clogging in the battery cathode. Both phenomena, limited electric conductivity of discharge products deposited and pore clogging, might appear for Na/O_2_ battery operation as well and could significantly affect battery performance and cycling stability.

While it was indicated that cubic particles of discharge product form on top of the cathode, as shown with scanning electron microscope (SEM) analysis^4^,^5^, the morphology of discharge product deep inside the cathode layers and the impact of current density on deposition behavior is not clear up to now. Recent studies have shown that X-ray tomography is highly suitable to monitor the discharge product in batteries in operando^23^,^24^,^25^,^26^. Moreover, imaging methods are suitable to resolve phases contributing to electrochemical processes, and degradation inside battery electrodes with high spatial resolution^27^,^28^.

In this work, we apply synchrotron X-ray tomography on cathodes of Na/O_2_ batteries with the aim to elucidate the spatial distribution of the discharge product within the cathode. We extend these measurements by SEM analysis of the formed particles on top of the cathodes of fully discharged batteries: Several Na/O_2_ batteries were discharged at a wide range of current density (50 to 1,000 *μ*A · cm^−2^) to visualize the impact of current density on discharge product deposition. We will discuss the distribution of NaO_2_ particles with regard to diffusivity and solubility of Na^+^ and the reactant O_2_ in the non-aqueous electrolyte applied. This analysis is dedicated to gain further understanding of the recently explored, reversible cell chemistry of Na/O_2_ batteries.

## Results

### Impact of current density on particle morphology

**Figure 1:**
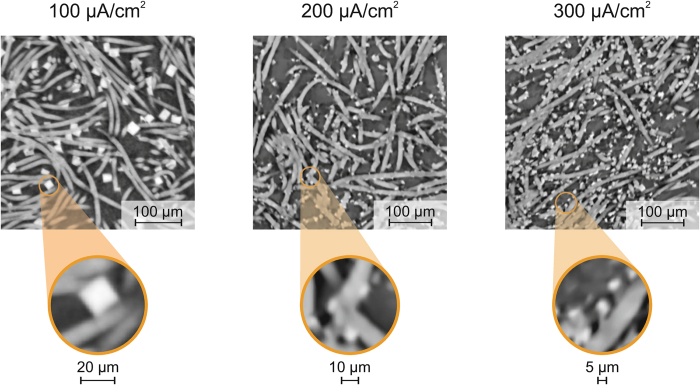
X-ray synchrotron tomography results: In-plane cross sections of selected areas showing the top (from O_2_ reservoir) of the cathodes analyzed for the current density indicated; discharge capacity 1.5 mAh; discharge product NaO_2_ (white color), fibers of the carbon cathode (gray color) and pore space (black color).

Figure 1 depicts in-plane cross sections of the carbon cathodes discharged at the respective current densities viewing on top of the battery from the O_2_ reservoir. For each cathode shown, the discharge product NaO_2_ (white particles) is deposited around the fibers of the carbon cathode (gray color). Pore space, i.e. filled with gas, is indicated with black color. At 100 *μ*A · cm^−2^ large (above 10 to 40 *μ*m maximal edge length) particles are visible, whereas at 200 *μ*A · cm^−2^ small (≈10 *μ*m maximal edge length) particles are present, and at 300 *μ*A · cm^−2^ even smaller (below 10 *μ*m maximal edge length) particles are formed.

**Figure 2:**
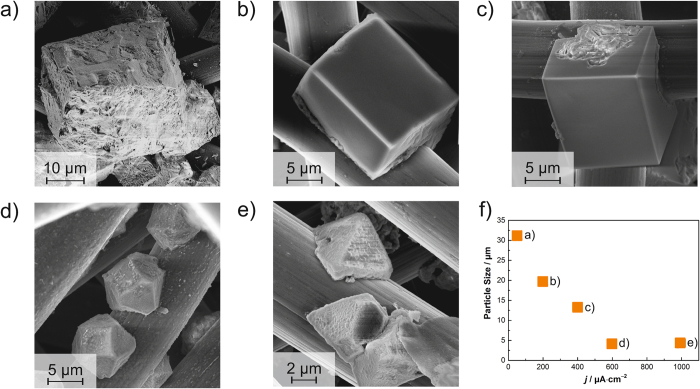
Scanning electron microscopy images from *ex situ* analysis of cathodes of fully discharged batteries, showing the discharge product NaO_2_: (**a**) 50 *μ*A · cm^−2^; (**b**) 200 *μ*A · cm^−2^; (**c**) 400 *μ*A · cm^−2^; (**d**) 600 *μ*A · cm^−2^; (**e**) 1,000 *μ*A · cm^−2^; (**f**) Particle size (maximal edge length) measured in the image as a function of discharge current density *j*.

To extend the aforementioned observations of the particle size for a wide range of current densities, SEM analysis was performed on cathodes from batteries that were galvanostatically discharged at 50, 200, 400, 600 and 1,000 *μ*A · cm^−2^, respectively, until a cut-off voltage of 1.8 V was reached. The respective discharge curves with the discharge capacity indicated are shown in the supplementary information in Fig. SI2. The SEM images obtained are shown in Fig. 2(a–e). We observe crystallized discharge product in form of cubic or pyramidal particles deposited on the carbon fibers for all cathodes analyzed. The particle size of all cubic particles decreases almost linearly from approximately 30 *μ*m to 5 *μ*m maximal edge length with increasing current density, which is illustrated in Fig. 2(f).

It is to be noted that the morphology of the discharge product changes at 600 *μ*A · cm^−2^: The cubic shape obtained for low current densities (Fig. 2(a–c)) changes to cuboctahedral shape (Fig. 2(d)) and then to pyramidal shape (Fig. 2(e)) at highest current densities.

### Discharge product distribution

**Figure 3:**
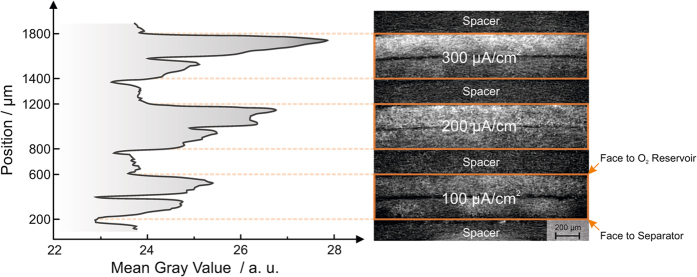
Mean gray values of through-plane projection of the battery cathodes analyzed with X-ray synchrotron tomography (left) and obtained images of the analyzed cathode stack (right). Each cathode is thereby approximately 2 × 200 *μ*m thick. Pristine carbon gas diffusion layers are placed as spacer between the cathodes.

Figure 3 depicts the stack of cathodes that was analyzed with synchrotron X-ray tomography in a projected through-plane view through the cathode centers. This view is used to visualize the distribution of mean X-ray beam attenuation in each cathode along its thickness with mean gray values in arbitrary units: Black colors represent attenuation due to a gas phase, gray colors represent attenuation due to the carbon fibers of the gas diffusion layer, and white colors represent high attenuating regions, i.e. the mean value of attenuation due to discharge product (NaO_2_) together with carbon fibers. Assuming a one-electron transfer reaction for the NaO_2_ formation, the local charge distribution in Na/O_2_ cells can be derived from this figure.

It can be seen that on top of all cathodes (facing the O_2_ reservoir), predominantly the discharge product NaO_2_ (white color, 25–28 a.u.) is present. At the bottom of the cathodes (facing the separator), less discharge product is found (lower gray values, 24–25 a.u.). The distribution of gray values along the gas diffusion layer thickness at 100 *μ*A · cm^−2^ appears to be more homogenous than for 200 than for 300 *μ*A · cm^−2^. The local minimum of gray values in the middle of each battery cathode can be explained as follows: Each cathode comprises of two 200 *μ*m thick gas diffusion layers that are stacked together in the battery housing (compare methods section). Consequently, less discharge product was deposited at the interface of both gas diffusion layers due to increased contact resistance.

### Particle size distribution

**Figure 4:**
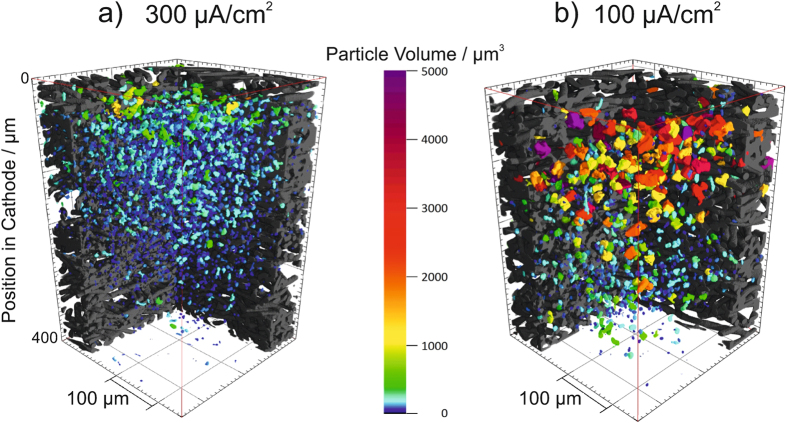
Particle size distribution analysis of selected regions (each time 1.70 × 1.70 × 0.45 mm^3^) inside the cathodes, showing the discharge product NaO_2_ and its volume at: (**a**) 300 *μ*A · cm^−2^; (**b**) 100 *μ*A · cm^−2^.

The high spatial resolution and contrast of synchrotron X-ray tomography enables to separate carbon fibers of the cathode and the reaction product NaO_2_ in the images obtained, and thus to analyze the particle size distribution (PSD) in selected regions. The respective representative results of the PSD analysis for 300 *μ*A · cm^−2^ and 100 *μ*A · cm^−2^ are visualized in Fig. 4(a,b), and in Fig. SI3 in the supporting information.

Small particles (particle volume below 1,000 *μ*m^3^, green and blue color) are present in the entire cathode from top to bottom of the battery that was discharged at 300 *μ*A · cm^−2^, whereas much larger particles (particle volume above 1,000 *μ*m^3^, orange/red and purple color) have formed at 100 *μ*A · cm^−2^. For 300 *μ*A · cm^−2^, particles with largest volume are located in the top of the cathode, whereas less particles are located in the bottom of the cathode. Thus, a slight gradient of particle volume from top to bottom of the cathode can be observed. All in all, at the top of the cathode, facing the O_2_ reservoir, more and larger particles are situated. For 100 *μ*A · cm^−2^, a more pronounced, steeper, gradient from top to bottom of the cathode from largest particles (≈3,000 *μ*m^3^) to smaller volume particles (≈1,000 *μ*m^3^) is visible. As for the cathode discharged at 300 *μ*A · cm^−2^, more large particles are present at the top of the cathode.

Furthermore, the PSD analysis of the entire sample region of the cathode (area of 2.5 × 2.5 mm^2^, compare Fig. SI4 in the supporting information) reveals that the number of particles is ~350,000 at 300 *μ*A · cm^−2^, which is much larger than for 100 *μ*A · cm^−2^ yielding ~80,000 particles, although both possess the same discharge capacity.

## Discussion

The PSD analysis and the gray value distribution yield that the discharge product in Na/O_2_ batteries is not homogeneously spread deep down into the cathode although all cathodes investigated are from batteries with 1.5 mAh discharge capacity. In detail, a gradient of discharge product can be observed along the cathode dimensions: NaO_2_ particles are predominantly deposited at the side of the O_2_ reservoir. By implication, the local charge density distribution in Na/O_2_ cells is higher near the O_2_ reservoir. This might be due to the limited solubility and low diffusivity of O_2_ in the electrolyte (see Hartmann *et al.*^8^). This hinders the transport of O_2_ to carbon fibers near the separator, where O_2_ should react with Na^+^ to form NaO_2_. The ionic conductivity of Na^+^ in the electrolyte is not the limiting factor for the deposition of NaO_2_, since particles are also formed at elevated current densities of 300 *μ*A · cm^−2^ on top of the cathode (compare Fig. 1), and for 400 to 1,000 *μ*A · cm^−2^ inside the carbon fibers on top of the cathode (compare Fig. 2), which is furthest away from the source of Na^+^, i.e. the metal anode.

It appears that the space occupied by discharge product strongly depends on the current density applied: The cathode is homogeneously utilized at low current densities, whereas more of the discharge product might be located near the O_2_ reservoir for elevated current densities (compare gradient of gray values in Fig. 3). By implication, structured cathodes with a porosity gradient (higher porosity facing the O_2_ reservoir, lower porosity facing the separator) might be necessary for Na/O_2_ batteries to obtain a homogenous discharge product distribution also at high current densities.

We furthermore deduce that the particle size of the discharge product directly scales with the current density applied: at 100 *μ*A · cm^−2^ discharge product of cubic shape (above 10 *μ*m edge length, compare Figs 1 and 2) forms, which is in line with other *ex situ* SEM image results^3^,^29^ and first-principle studies^30^.

Considering that the discharge product NaO_2_ is an insulator and that its formation and decomposition during cell cycling was identified as a solution-based process^31^, another conclusion is derived in the following from the tomography results obtained: At elevated current densities, very small NaO_2_ particles form around the carbon fibers in a film-like manner (compare Fig. 1). Even if sufficient Na^+^ conductivity is ensured, electrons might not reach the reaction zone at the interface of solid and electrolyte due to poor electrical conductivity of the reaction product deposited. Thus, it seems that only capacities below 0.5 mAh can be discharged above 200 *μ*A · cm^−2^ (compare Fig. SI2 and Hartmann *et al.*^29^). Due to the poor electric conductivity of NaO_2_ particles, electron transport to the electrode/electrolyte interface might be limited, and cell potential decays rapidly. By implication, small particles of discharge product can decrease the available battery capacity significantly by surface blocking. Whereas on the other hand, large particles might cause pore clogging, and thus cause O_2_ shortage, as indicated by the three-dimensional representation of the cathode discharged at 100 *μ*A · cm^−2^ shown in Fig. 4(b). To overcome this issue, either pumping of electrolyte (electrolyte flow battery) or active supply of oxygen might be a valid option for Na/O_2_ battery operation. Moreover, fluorinated ether might be added to the liquid electrolyte, for example as found for Li/O_2_ batteries^32^, to enhance oxygen activity and thus to achieve a homogenous distribution of NaO_2_ particles in the cathode. In addition, the use of redox mediators (dissolved in the liquid electrolyte^33^,^34^) might help to achieve a more homogenous distribution of discharge product and to assist the decomposition of NaO_2_ particles during charge.

All in all, our analysis yields comprehensive insight into the spatial distribution of discharge product in the battery cathode of Na/O_2_ batteries and will help to understand limitations for the operation at elevated current densities that arise thereof, the cell design chosen, and the operation mode selected. It might be for example beneficial to consider new operation strategies for Na/O_2_ batteries to achieve a more uniform distribution of discharge product. The analysis presented will help to systematically identify further limitations for Na/O_2_ batteries, which in the end might help to improve their capacity and cycle life. Other research fields (e.g. the model-based analysis of cathode processes and cell concepts^35^,^36^) might benefit from the here presented findings.

## Methods

### Battery preparation and discharge

**Figure 5:**
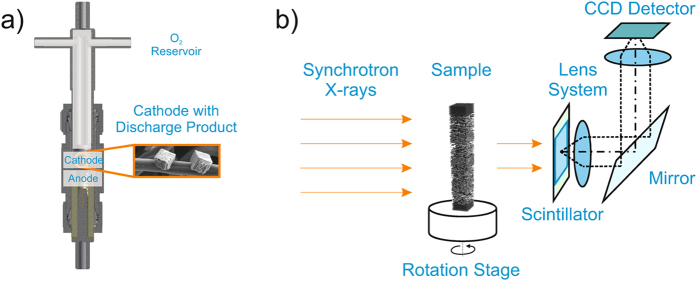
(**a**) CAD-drawing of the Swagelok design with two electrode setup for electrochemical measurements (cathode gas diffusion layer indicated in the inset); (**b**) Schematic: Synchrotron X-ray tomography of the disassembled cathodes filled with discharge product.

Electrochemical measurements were performed using the ‘Giessen cell’^3^,^29^, a cell based on a Swagelok design with two electrodes that is illustrated in Fig. 5(a). Two binder-free gas diffusion layers type H23 with 200 *μ*m thickness and diameter of 12 mm each (Freudenberg, Weinheim, Germany) were used as cathode, and pure sodium metal (BASF SE, Ludwigshafen, Germany) was used as anode (12 mm diameter). One glass microfiber filter (GF/A, Whatman, 12 mm diameter) separated the electrodes. Electrolyte comprising of diglyme (anhydrous, 99.5% Sigma Aldrich) as solvent and 0.5 M sodium triflate (NaSO_3_CF_3_, 98%, Aldrich) as conducting salt was applied on gas diffusion layer and separator. The cell design comprises an oxygen reservoir, which was flushed with oxygen (purity 5.0, Praxair) for 10 seconds at 10^5^ Pa just before the electrochemical measurement. All cells were assembled in an Ar-filled glove box (GST4, Glovebox Systemtechnik) with water and oxygen contents below 5 ppm.

Three cells of aforementioned composition were galvanostatically discharged at 100, 200 and 300 *μ*A · cm^−2^ in a temperature chamber (Binder) at 298 K with a battery cycling system 4300 (Maccor) until a discharge capacity of 1.5 mAh was reached, to achieve comparable loading with discharge product. The corresponding discharge curves are shown in Fig. SI1 in the supporting information. After discharge, these batteries were disassembled in the Ar-filled glove box. 2.5 mm diameter samples of the respective cathodes (compare Fig. SI4 in the supporting information) were cut out and put all together in a PEEK sample holder, sealed with hot glue, and then analyzed with synchrotron X-ray tomography as visualized in Fig. 5(b). The stacking of the cathodes analyzed is illustrated in Fig. 3.

For SEM imaging analysis, batteries of aforementioned composition were discharged with the ‘Giessen cell’ setup with three electrodes with the aforementioned battery cycling system at 50, 200, 400, 600 and 1,000 *μ*A · cm^−2^, respectively (see supplementary information, Fig. SI2) and their cathodes were analyzed *ex situ* with SEM, viewing on top of the cathode (i.e. from the O_2_ reservoir).

### Imaging

Synchrotron X-ray tomography imaging was performed at the synchrotron tomography station of the Helmholtz-Zentrum Berlin (BAMline at Bessy II). The respective setup is illustrated in Fig. 5(b). To ensure sufficient transmission through the samples, a monochromatic X-ray beam with an energy of 15 keV was chosen. A 4,008 × 2,672 pixel^2^ CCD camera (PCO 4000 with a CdWO4 scintillator screen) was used to capture local radiograms over 360 degrees. Stitching always two 180 degree separated local radiograms to one radiogram together resulted in a 7,900 × 7,900 × 2,672 pixel reconstruction with a voxel size of 0.438^3^ *μ*m^3^. For the whole sample, two local measurements had to be performed and were stitched together with the software Fiji^37^.

A conventional X-ray tube was used for full view tomographic measurements of cathodes for the results in Fig. SI4 in the supporting information. The accelerating voltage was tuned to 50 kV while the current at the tungsten anode was adjusted to 200 *μ*A. No filter was applied. 1,500 projections were taken for a full tomography (full range over 360 degrees). Each angle step was exposed for 2.2 s three times to increase the signal-to-noise-ratio. The projections were taken using a Hamamatsu flat panel detector with 2,316 × 2,316 pixel with a pixel size of 50 *μ*m (6.25 *μ*m pixel size in the reconstructed two-dimensional images).

SEM measurements were performed with a Merlin high-resolution Schottky field emission electron microscope (Zeiss SMT) equipped with an X-Max EDS detector (Oxford Instruments). All analyzed cathodes were washed prior each SEM measurement to remove excess liquid electrolyte.

### Particle size distribution analysis algorithm

A sub stack that contained two slices of every of the cathodes analyzed was created in order to train a machine learning data-mining algorithm. For that purpose the trainable Weka Segmentation^38^ plug-in for Fiji^37^ including a fast random forest classifier was used. The classifier was trained by means of the sub stack. Subsequently, the segmentation of the full data set was performed by adapting the classifier to this data set. Finally the segmented data set was labeled using the software Mavi 1.5.1^39^ and was analyzed with the software Avizo Fire 8.0^40^. The three-dimensional visualization in Fig. 4 of the segmented data was created using VGStudio MAX 2.2.6^41^.

## Additional Information

**How to cite this article**: Schröder, D. *et al.* Visualizing Current-Dependent Morphology and Distribution of Discharge Products in Sodium-Oxygen Battery Cathodes. *Sci. Rep.* **6**, 24288; doi: 10.1038/srep24288 (2016).

## Supplementary Material

Supplementary Information

Supplementary Movie 1

Supplementary Movie 2
