# Supplementary material for: Visualizing Current-Dependent Morphology and Distribution of Discharge Products in Sodium-Oxygen Battery Cathodes
Source: Sci Rep. 2016 Apr 12;6:24288. doi: 10.1038/srep24288 (PMC4828675; doi:10.1038/srep24288)
Supplement: Supplementary Information [file srep24288-s1.pdf]

Supplementary Information: Visualizing Current-Dependent Morphology and Distribution of Discharge Products in Sodium-Oxygen Battery Cathodes

Daniel Schröder[1,*], Conrad L. Bender[1], Markus Osenberg[2], André Hilger[2], Ingo Manke[2], and Jürgen Janek[1]

[1]Physikalisch-Chemisches Institut, Justus-Liebig-Universität Giessen, Heinrich-Buff-Ring 17, 35392 Giessen, Germany
[2]Helmholtz-Zentrum Berlin für Materialien und Energie GmbH, Hahn-Meitner-Platz 1, 14109 Berlin, Germany
[*]daniel.schroeder@phys.chemie.uni-giessen.de

ABSTRACT

Discharge Curves

Discharge curves for batteries investigated with synchrotron X-ray tomography are shown in Fig. SI 1. Discharge curves for batteries investigated with SEM are shown in Fig. SI 2.

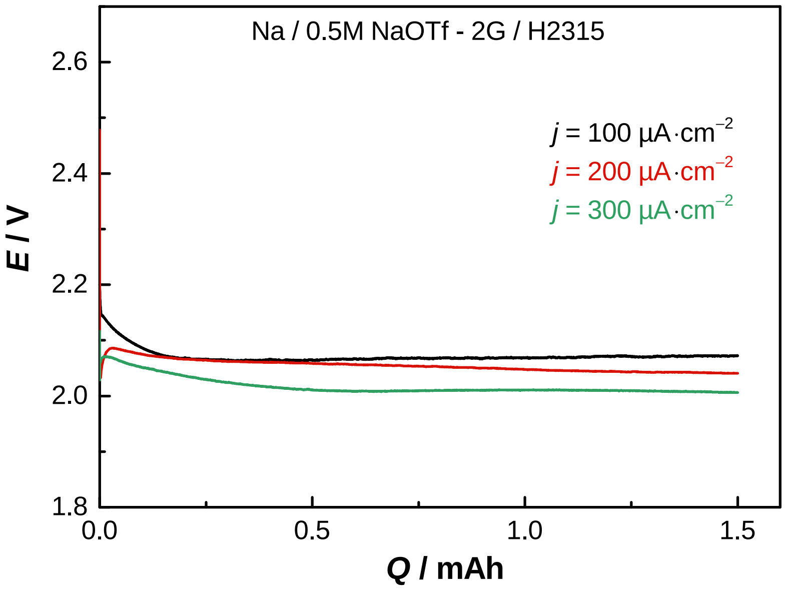

Fig. SI 1. Discharge curves for three different batteries analyzed with ex situ synchrotron X-ray tomography; discharge at various current densities until a capacity of 1.5 mAh was reached.

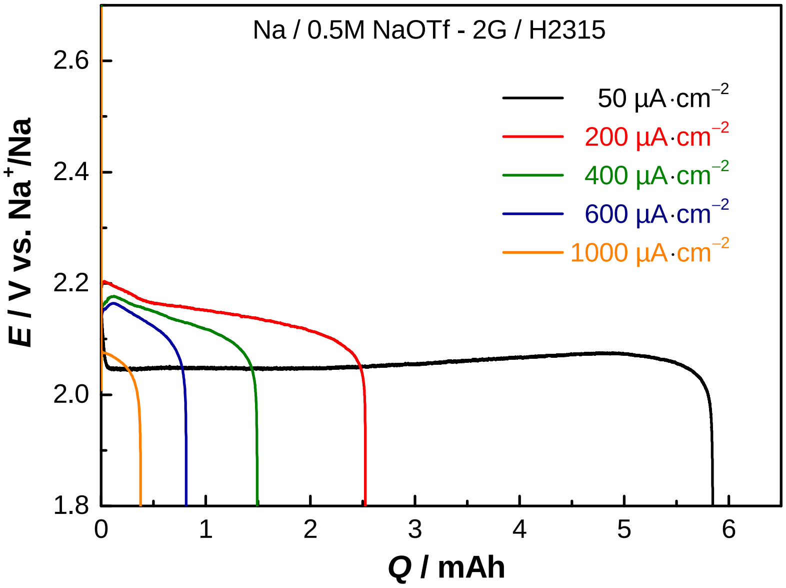

Fig. SI 2. Discharge curves at constant current density of the Na/O_2 batteries that were analyzed with scanning electron microscopy.

Particle Size Distribution - Further Results

Fig. SI 3 reveals the distribution of particle volume inside the cathode from top to bottom, including the count of particles indicated by color value for 300 $\mu A \cdot cm^{-2}$ and 100 $\mu A \cdot cm^{-2}$, respectively.

Inhomogeneous Distribution in the Cathode

Fig. SI 4 depicts a full view maximum projection of a cathode analyzed with a conventional X-ray tube (i.e. μCT analysis). This analysis is used to visualize possible inhomogeneities of the discharge product in the cathode. It can be observed that higher attenuation areas (white color), i.e due to discharge product, are not evenly distributed through the entire cathode. In particular, circular regions (light blue arrows) with high attenuation are visible, which suggests that distribution of discharge product in x-direction and y-direction of the cathode in Na/O_2 cells is rather inhomogeneous. This is probably due to the application of perforated current collectors in the 'Giessen cell' (compare Figure S6 in the supporting information of Bender et al.[1]). The perforated current collectors seem to enhance oxygen transport only in certain areas, so that discharge product is preferably formed near the void spaces of the perforated current collector. This finding implies that the samples cut out for the ex situ synchrotron X-ray analysis (see Fig. 1 and Fig. 4 in the manuscript) also contain the here observed inhomogeneous distribution of discharge product.

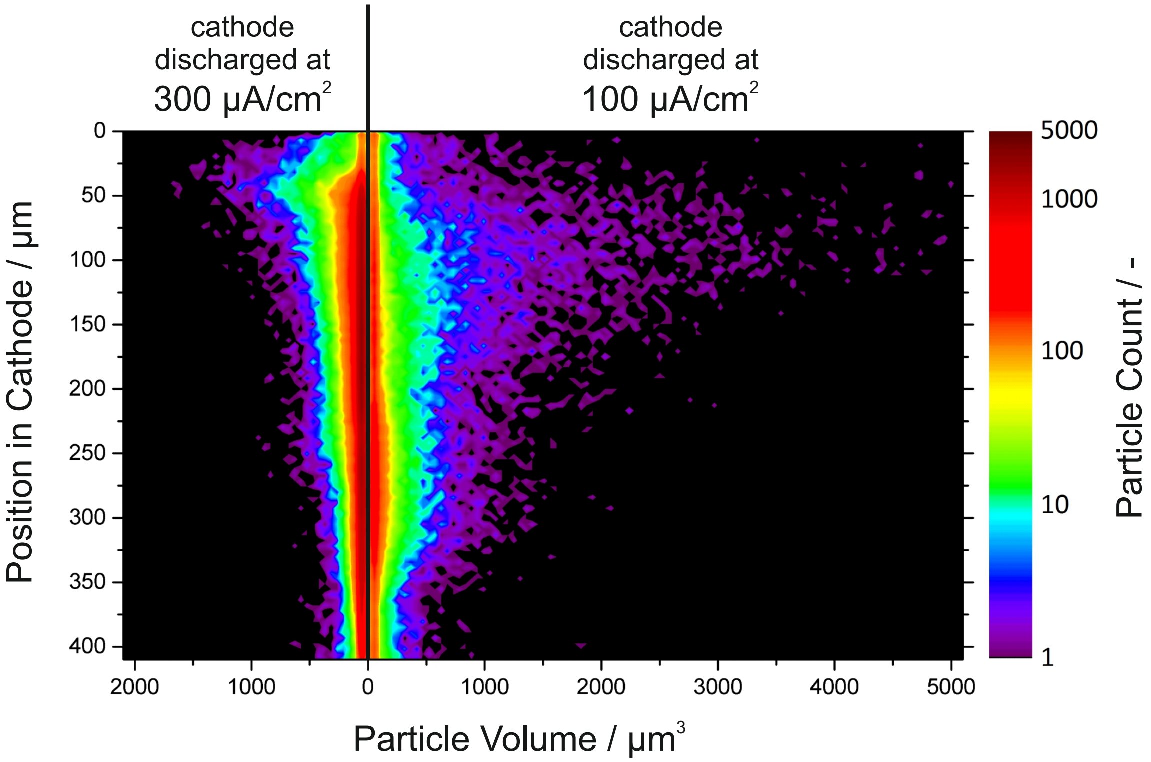

Fig. SI 3. Particle volume inside each cathode from 0 μm (O_2 reservoir) to 400 μm (separator side) with respective count of particles indicated; particle count as presented in Fig. 4 in the main part of the manuscript.

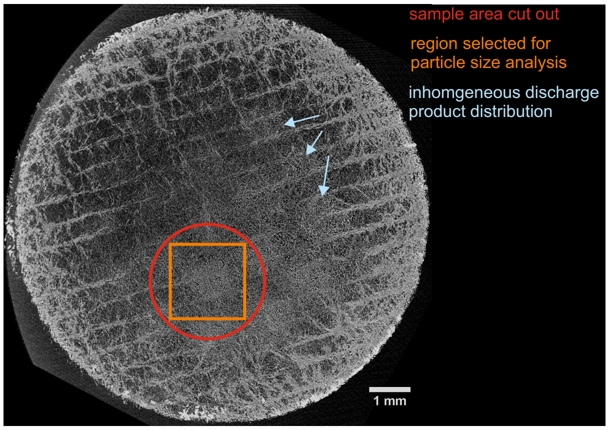

Fig. SI 4. Top view of a maximum projection (accumulated for all slices, 200 μm thick) of a battery cathode discharged at 100 $\mu A \cdot cm^{-2}$. Imaging obtained with μCT analysis as described in the methods section. White areas represent high attenuating phases (i.e. filled with product particles), darkest gray areas represent the carbon fibers of the cathode and black areas indicate gas phase. Additionally, we indicated the sample area (2.5 x 2.5 mm^2) that was cut out for ex situ synchrotron X-ray analysis and the region selected for the particles size distributions analysis (1.70 x 1.70 mm^2).

Videos of Particle Size Distribution

Videos of NaO_2 particles in the cathodes investigated are shown in the online supplementary information.

References

1. Bender, C. L., Hartmann, P., Vračar, M., Adelhelm, P. & Janek, J. On the thermodynamics, the role of the carbon cathode, and the cycle life of the sodium superoxide (NaO2) battery. *Advanced Energy Materials* **4**, 1–10 (2014).
